# Improving Mechanical Properties and Reaction to Fire of EVA/LLDPE Blends for Cable Applications with Melamine Triazine and Bentonite Clay

**DOI:** 10.3390/ma12152393

**Published:** 2019-07-26

**Authors:** Guadalupe Sanchez-Olivares, Antonio Sanchez-Solis, Octavio Manero, Ricardo Pérez-Chávez, Mario Jaramillo, Jenny Alongi, Federico Carosio

**Affiliations:** 1 CIATEC, Asociación Civil, Omega 201, Colonia Industrial Delta, León 37545, Gto., Mexico; 2 Instituto de Investigaciones en Materiales, Universidad Nacional Autónoma de México, Avenida Universidad 3000, Ciudad de México 04510, Mexico; 3 Dipartimento di Chimica, Università degli Studi di Milano, Via Golgi 19, 20133 Milano, Italy; 4 Dipartimento di Scienza Applicata e Tecnologia, Politecnico di Torino-Alessandria Campus, Viale Teresa Michel 5, 15121 Alessandria, Italy

**Keywords:** EVA/LLDPE blend, flame retardant, wire and cable, melamine triazine, clay

## Abstract

The high flame-retardant loading required for ethylene-vinyl acetate copolymer blends with polyethylene (EVA-PE) employed for insulation and sheathing of electric cables represents a significant limitation in processability and final mechanical properties. In this work, melamine triazine (TRZ) and modified bentonite clay have been investigated in combination with aluminum trihydroxide (ATH) for the production of EVA-PE composites with excellent fire safety and improved mechanical properties. Optimized formulations with only 120 parts per hundred resin (phr) of ATH can achieve self-extinguishing behavior according to the UL94 classification (V0 rating), as well as reduced combustion kinetics and smoke production. Mechanical property evaluation shows reduced stiffness and improved elongation at break with respect to commonly employed EVA-PE/ATH composites. The reduction in filler content also provides improved processability and cost reductions. The results presented here allow for a viable and halogen-free strategy for the preparation of high performing EVA-PE composites.

## 1. Introduction

The need for safe materials represents one of the main driving forces continuously pushing the developments and advances in materials science and technology. The area of fire retardant materials is of great concern. Indeed, recent catastrophic events and present legislations clearly highlight the potential danger related to fire events, as well as the environmental and toxicological risks associated with some of the most commonly used flame-retardant chemicals. Particular fire risk is associated with electrical cables as they contain several polymeric parts (insulation, bedding, sheath) constituting fuel sources (for fire start and spread) as a consequence of arcing, excessive ohmic heating (without arcing) and external heating [1,2]. Ethylene-vinyl acetate copolymer (EVA) and EVA blends with polyethylene (EVA-PE) are among the most widely used polymers for insulation and sheathing of electric cables. Common practice is to load the polymer with high amounts (typically 60–70 wt.%) of metal hydrates such as aluminum hydroxide or magnesium hydroxide [3]. Another possible strategy to more efficiently reduce the fire and environmental risks of these materials comprises the use of nanoparticles in combination with halogen-free flame-retardant additives (FRs) [4,5]. For instance, the fire retardancy of ethylene-vinylacetate (EVA) and low-density polyethylene (LDPE) blends using organoclay in combination with either aluminum or magnesium hydroxide has been assessed by thermogravimetric analysis and cone calorimetric measurements evaluating the effect of the surface layer formed during pyrolysis of the polymer nanocomposites by numerical models [6]. The inclusion of 68 wt.% of metal hydroxides considerably reduced the heat release rate (HRR); this effect was further improved by coupling the hydroxide with 5 wt.% of organoclay. The reduction of HRR was attributed to the formation of a ceramic-like layer on the surface of unpyrolysed material in the solid phase during pyrolysis. This ceramic-like layer acts as a barrier reducing the heat and mass transfer thus resulting in a flame retardant effect. Remarkable improvements on flame retardancy by combinations of nanofiller and aluminum trihydroxide (ATH) were found in EVA [7]. These performances have been correlated to the formation of a more efficient protective barrier during combustion, thus, highlighting the beneficial role of the inclusion of relatively small amounts (≤5 wt.%) of nanofiller. 

Similar improvements have been observed in EVA blends for industrial cable applications by including modified bentonite clay and ATH; however, while significantly contributing to the flame retardancy properties, the presence of the nanofiller did not result in improvements in the tensile strength and elongation at break of prepared composites [8]. The detrimental effects on mechanical properties linked to the high FR loading required for the cable application of EVA and EVA blends is a well-known issue [9].

Indeed, due to the high loading required to obtain suitable FR properties for cable applications, the resulting materials are more rigid and brittle than the unmodified polymer and thus more prone to damage and cracking, eventually reducing the insulation and protection of the polymer sheath [10,11]. In order to solve this problem, research aims at the development of new and environmentally friendly flame-retardant systems capable of being efficient at loading levels below traditional amounts. Nitrogen-based compounds, such as triazines, represent a possible candidate to be incorporated in a FR system with improved efficiency [12,13]. As an example, the combination of ammonium polyphosphate and a commercial triazine derivative (poly-[2,4-(piperazine-1,4-yl)-6-(morpholine-4-yl)-1,3,5-triazine]/piperazine) has been adopted to produce a novel phosphorous nitrogen intumescent flame-retardant system for polypropylene capable of granting self-extinguishing properties at low FR content (i.e., 20 wt.%) [14]. The use of triazine derivatives as part of an intumescent system has proven to provide good effects on the fire retardancy performances of polymers [15]. However, such intumescent systems are relatively expensive and their application for electrical requirements is rather limited. In the present paper, melamine triazine has been used in combination with bentonite nanoparticles and aluminum hydroxide to produce a novel FR system for EVA-PE blends, capable of achieving excellent flame-retardant performance while preserving their mechanical properties. The developed FR systems allow for a reduction of the filler loading down to 37% with improved mechanical properties while granting FR performance suitable for electrical cables applications. This work provides a viable solution for the preparation of FR EVA-PE blends with reduced costs and improved efficiency.

## 2. Materials and Methods 

**Figure 1:**
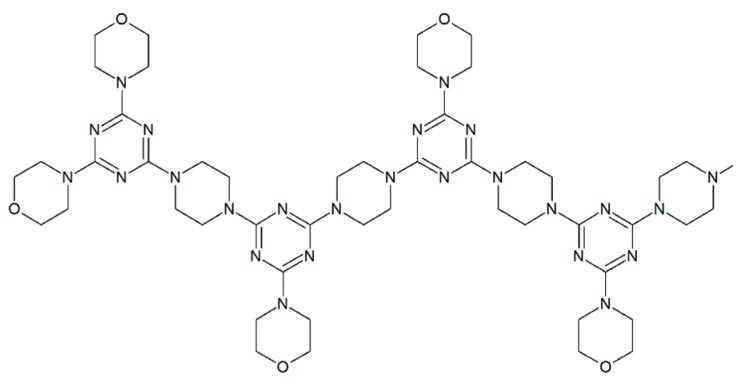
Chemical formula of melamine triazine.

Elvax^®^ 460, a copolymer of ethylene vinyl acetate with 18 wt.% vinyl acetate content (2.5 g/10 min melt flow rate) from Dupont and BDL 92010 C, and linear low density polyethylene (LLDPE) (1.0 g/10 min melt flow rate) from PEMEX-Petroquímica, Coatzacoalcos, Ver., Mexico were purchased. Fusabond N493, an anhydride-modified ethylene copolymer (1.6 g/10 min melt flow), supplied by Dupont, was employed as compatibilizer. ALOLT 60DLS, aluminum trihydroxide (ATH), with average particle size d_50_ of 1.0–2.2 microns (99.5% purity and surface area of 12 m^2^/g) was purchased from Mal Hungarian. MCA^®^ PPM Triazine HF, melamine triazine (TRZ), poly-[2,4-(piperazine-1,4-yl)-6-(morpholine-4-yl)-1,3,5-triazine]/piperazin (Figure 1), was supplied by MCA Technologies GmbH. ATH and TRZ were used as halogen-free FRs. Actisil 220FF, sodium bentonite clay (55 meq/100g cationic capacity), was purchased from Clariant. Sodium bentonite clay was modified using *L*-lysine mono-chlorohydrated via ionic interchange reaction, as previously reported [16,17,18]. Briefly, 100 g of sodium bentonite clay was added to a water solution of 10 g *L*-lysine in 1.5 L. After 30 min stirring, the suspension was decantated and the solid was retrieved by filtration and drying of the modified bentonite clay (L-lysine loading = 3 wt.%). 

The adopted EVA-PE blend was composed of 67 parts of EVA, 17 parts of LLDPE and 16 parts of Fusabond N493 [19,20,21]. This formulation constitutes the polymer matrix (EVA/LLDPE/compatibilizer) coded as E-PE. The polymer composites containing ATH, TRZ and clay were obtained in a co-rotating twin-screw extruder type SHJ 40D, with an optimized configuration for easier compounding and dispersing process as demonstrated elsewhere [22]. The extruder had a 41-mm diameter and a length/diameter ratio = 40, and 10 independent heating zones for optimal processing. A detailed description of the twin-screw configuration is reported in the Supporting Information file. Table 1 summarizes the composition of investigated composites.

The extrusion process was carried out at 300 rpm rotational speed with a temperature profile of 137/137/157/157/165/170/175/180/185/185 °C, from feeding zone to die (Figure S1). 

Specimens for flammability, cone calorimetry and mechanical tests were produced by injection molding in a Milacron M50 machine (Milacron LLC, Karnataka, India) at 170/175/180/180 °C temperature profile, 70 mm/s injection/fill speed, 110 bar pack/hold pressure, 15 s pack/hold time and 20 s cooling time.

The morphology of E-PE blends and the corresponding composites was studied using a field-emission scanning electron microscope (SEM) JEOL JSM-7600F (JEOL, Ltd., Akishima, Japan). Specimens were prepared by using cryogenic fragile fracture and gold-coated prior to SEM analyses. Element mapping was carried out in the scanned area by energy dispersive spectroscopy (EDS, Oxford Instruments, Concord, MA, USA). Thermal stability was evaluated by thermogravimetric analysis by a TA-Instrument Q-550 equipment (TA-Instrument, Inc., New Castle, DE, USA) at a heating rate of 10 °C/min from 25 °C up to 800 °C, under argon and air atmospheres. The sample size was 10 ±1 mg, the experimental error was ±1 °C and ±0.01 wt.%. Flammability was assessed following UL94 vertical classification tests according to the ASTM D3801-19 standard [23], using specimens with dimensions of 127 × 12.7 × 3.1 mm^3^. Combustion behavior under forced combustion was investigated by cone calorimetry (Fire Testing Technology). Specimens (100 × 100 × 3 mm^3^) were exposed to a 35 kW/m^2^ radiative heat flux in horizontal configuration. Average values concerning time to ignition (TTI), peak of heat release rate (pkHRR), total heat release (THR), maximum average rate of heat emission (MARHE), total smoke release (TSR) and final residue were evaluated and are presented with their experimental deviations. Measurements were performed four times for each formulation. Prior to flammability and forced combustion tests, all specimens were conditioned in a climatic chamber (23 ±1 °C 50% relative humidity) for 48 h. Tensile tests were carried out using an Instron Universal testing machine 5565 model (Instron Corp., Norwood, MA, USA) at a crosshead speed of 50 mm/min at 25 ±2 °C and type I specimen dimensions, following the ASTM D638 standard [24]. At least five specimens were tested for each sample, and the average value for Young modulus, tensile strength, elongation at break and tenacity is reported. 

The rheological behavior of the composites was measured in a strain-controlled Ares G2 TA-Instrument (TA-Instrument, Inc., New Castle, DE, USA) rheometer using parallel plates of 25 mm diameter. All tests were performed at 195 °C under small amplitude oscillatory shear flow (SAOS). The dynamic frequency sweep mode was carried out in linear viscoelastic regimen with a strain of 1% from 0.1 to 100 rad/s.

## 3. Results and Discussion

### 3.1. Morphology

**Figure 2:**
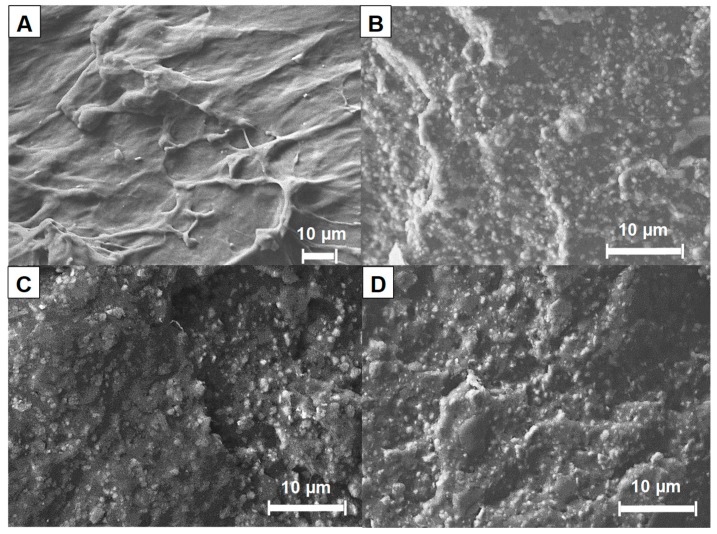
Scanning electron microscopy (SEM) micrographs of fractured surface for: (**A**) E-PE, (**B**) E-PE/185ATH, (**C**) E-PE/120ATH/15TRZ, and (**D**) E-PE/120ATH/15TRZ/3clay.

The morphology of the prepared composites was studied by scanning electron microscopy (SEM). Figure 2 displays SEM micrographs of the neat polymer matrix (E-PE blend, Figure 2A) and selected corresponding composites containing ATH, ATH/TRZ and ATH/TRZ/clay (Figure 2B,D).

**Figure 3:**
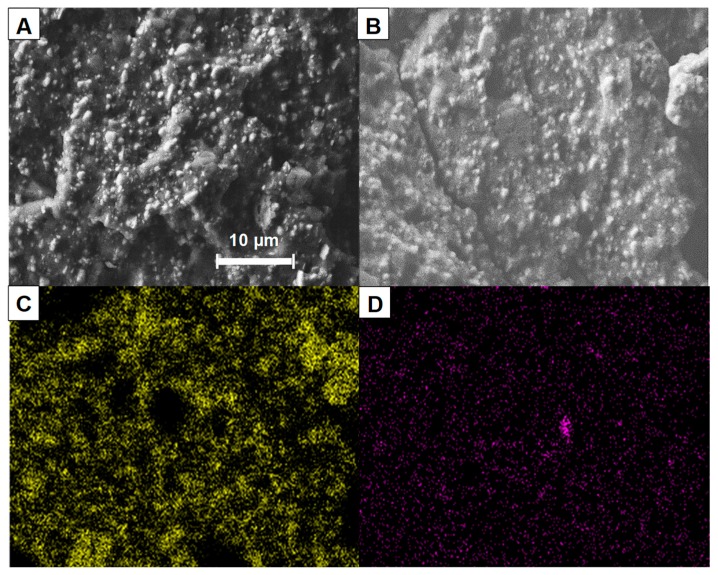
(**A**) SEM micrograph of fractured surface of E-PE/120ATH/10TRZ/5clay composite, (**B**) scanned area of E-PE/120ATH/10TRZ/5clay composite for elemental analysis, (**C**) aluminum and (**D**) Si mapping.

E-PE polymer blend shows a ductile fracture characterized by a deformed surface and a continuous pattern; no phase separation or droplets are observed for this blend (Figure 2A). This is ascribed to the use of the compatibilizer (anhydride-modified ethylene copolymer) as it is well-known that blends based on ethylene-vinyl acetate copolymer and polyethylene (i.e., LDPE or LLDPE) are immiscible [25]. The absence of a compatibilizer would result in phase separation and polymer droplet formation as a function of the interfacial tension and viscosity ratio between the EVA and PE [26]. The inclusion of ATH deeply modifies the resulting morphology in a fragile fracture attributed to the presence of the filler (Figure 2B). ATH particles, with dimensions ranging from sub micronic up to 4 μm, show a good distribution and dispersion within the polymer matrix even at such high filler loading (185 phr, 65 wt.%). This is ascribed to the screw configuration and high rotational speed employed during the melt extrusion process. A similar morphology is observed for composites containing TRZ and TRZ/clay with reduced ATH content (Figure 2C,D), thus highlighting no substantial changes in the distribution of ATH in the presence of the other additives. The distribution of clay within the composites was further evaluated by elemental mapping; Figure 3 reports the aluminum and silicon elemental analysis of E-PE/120ATH/10TRZ/5clay.

According to the silicon elemental analysis, clay particles exhibit a homogenous dispersion and distribution. No agglomerates are observed, likely due to the low clay content within the composites (i.e., 5 phr, 2 wt.%). Furthermore, Al distribution is similar to that of E-PE/120ATH (Figure S2) highlighting that the presence of TRZ and clay does not alter ATH distribution and dispersion.

### 3.2. Rheological Properties

**Figure 4:**
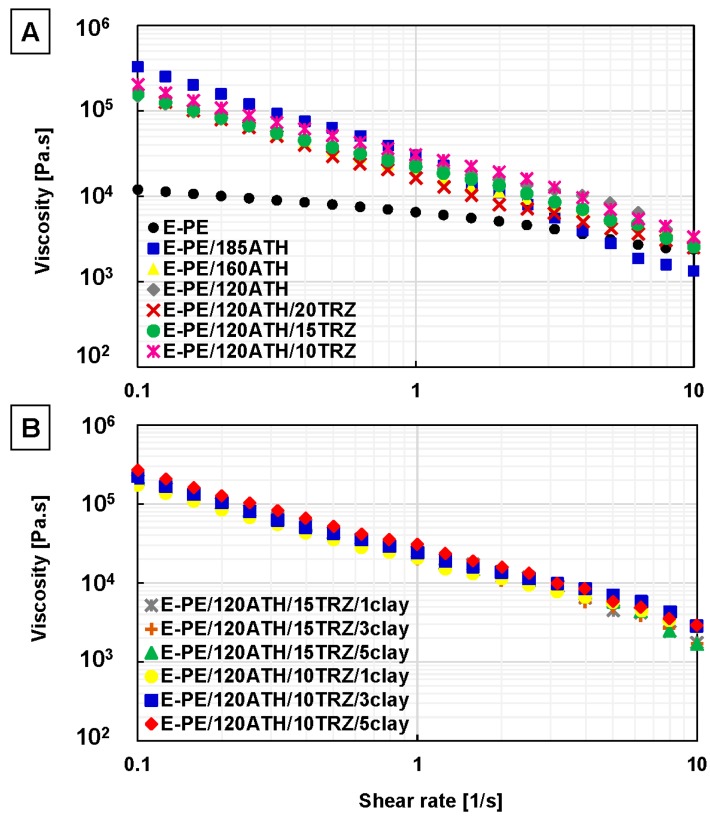
Simple shear viscosity as a function of shear rate for: (**A**) E-PE blend, E-PE/ATH and E-PE/120ATH/TRZ composites using ATH and TRZ at different content, and (**B**) E-PE/120ATH/15TRZ/clay and E-PE/120ATH/10TRZ/clay composites varying clay content.

Rheological properties of the E-PE blend and composites included continuous simple and small amplitude oscillatory shear flow. This test provides information on the dispersion of the filler within the polymer matrix. Figure 4 depicts shear viscosity as a function of shear rate for E-PE blend and investigated composites.

**Figure 5:**
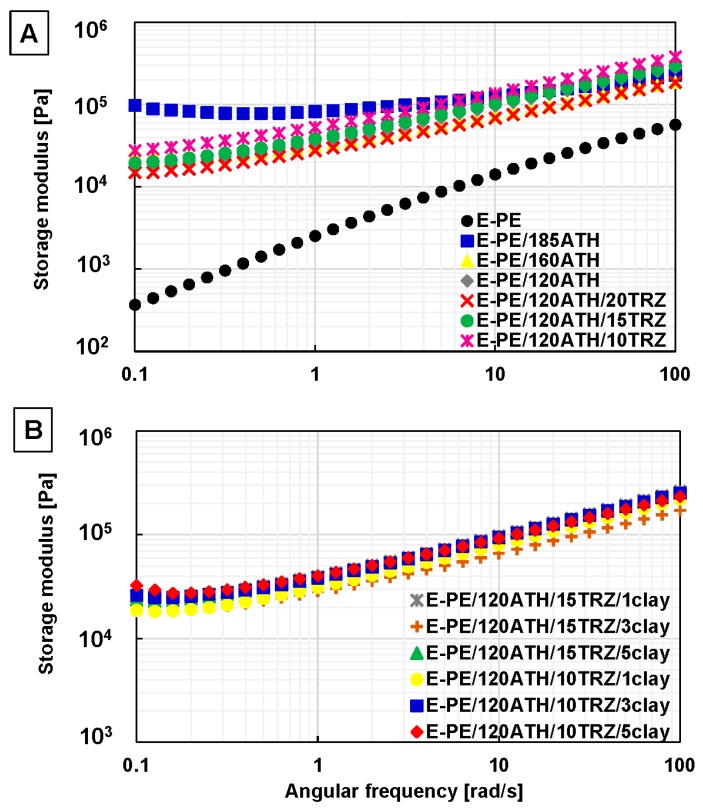
Storage modulus as function of angular frequency in small amplitude oscillatory shear flow (SAOS) flow test for: (**A**) E-PE blend, E-PE/ATH and E-PE/120ATH/TRZ composites using ATH and TRZ at different content, and (**B**) E-PE/120ATH/15TRZ/clay and E-PE/120ATH/10TRZ/clay composites varying clay content.

In Figure 4A, the shear viscosity of the polymer matrix (E-PE blend) presents a nearly Newtonian-like behavior at low shear rates (0.1–1.0 1/s). Newtonian-like behavior corresponds to the region in the flow curve where the viscosity becomes independent of the shear rate (i.e., a constant viscosity). On the other hand, E-PE blend presents moderate shear thinning behavior at high shear rate (1.0–10.0 1/s). By including ATH and TRZ in the formulation, the viscosity increases at low shear rates (0.1–1.0 1/s), reaching the maximum value for E-PE/185ATH. Nevertheless, at high shear rates (1.0–10 1/s) the viscosity of E-PE/185ATH composite decreases similarly to the rest of other formulations. All composites exhibit the typical feature of shear-thinning behavior with no plateau region observed over the studied shear rate range. Such behavior has been ascribed to a good dispersion of both flame retardants (ATH and TRZ) within the polymer matrix (Figure 4A) [27]. Composites containing clay show a remarkable shear-thinning behavior in the whole shear rate range (Figure 4B). Such behavior implies low shear viscosity at high shear rate, an important characteristic for the easy processing of these composites. In addition, the storage modulus was also evaluated, and its plot as function of angular frequency for E-PE blend and prepared composites is reported in Figure 5.

The neat E-PE blend displays a storage modulus with a constant slope over the entire frequency range, indicating non-terminal flow behavior characteristic of pure polymers [28]. Composites containing ATH and ATH/TRZ show a pronounced solid-like behavior at low frequency (0.1–1.0 rad/s). In particular, the storage modulus of E-PE/185ATH exhibited the highest value likely due to the high particle loading (Figure 5A). On the other hand, the presence of TRZ does not influence the storage modulus, as all TRZ containing composites disclose a similar slope in the whole frequency range with respect to E-PE/120ATH. This result indicates the absence of interactions between the filler particles in the flow stage. A similar behavior is observed for clay containing formulations (Figure 5B). The performed rheological measurements suggest a good dispersion of flame retardant additives within the polymer matrix, further confirming previous SEM observations.

### 3.3. Thermal Stability

The thermal stability of neat components and prepared composites under inert and oxidative atmosphere has been evaluated by thermogravimetric analyses in argon and air, respectively. As far as neat components are concerned (Figure S3 and Table S1), neat ATH yields a weight loss ascribed to its dehydration with consequent water release within a 200–300 °C range. Bentonite clay shows a slow and constant weight loss associated to its dehydration from the interlayer space and cavities, and cation hydration spheres, as well as dihydroxylation at high temperatures [29,30]. On the other hand, TRZ shows a more complicated degradation path associated to melamine gradual condensation releasing melam, melem and melon products due to ammonia elimination [31].

**Figure 6:**
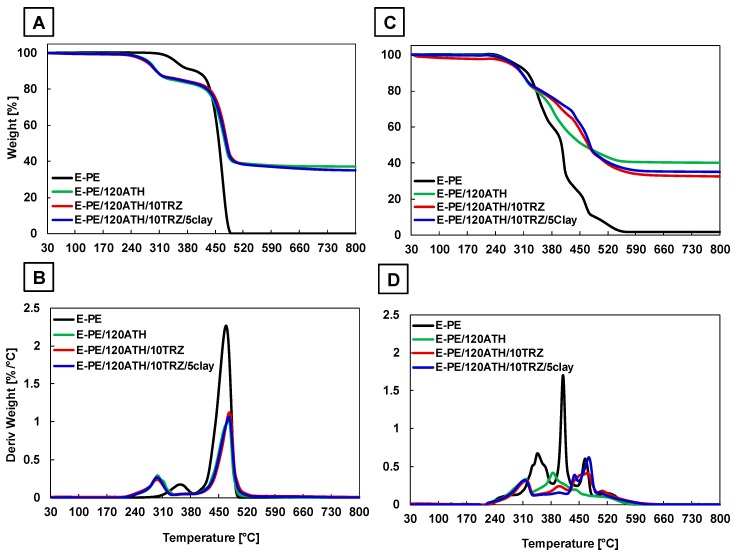
TG and dTG curves of E-PE, E-PE/120ATH, E-PE/120ATH/10TRZ and E-PE/120ATH/10TRZ/5clay composites. (**A**,**B**) curves in argon, and (**C**,**D**) curves in air atmospheres.

Figure 6 displays TG and dTG curves, and Table 2 discloses the collected data of the most representative samples. The complete curves and thermal data are reported in Figures S4–S6 and Tables S2–S4, respectively.

Under non-oxidative conditions, the E-PE blend decomposes in two steps (Figure 6A,B). The first occurs at nearly to 350 °C and corresponds to the de-acylation of the vinyl acetate groups in EVA [31,32]. The second step, associated with the highest weight loss, takes place between 400 and 500 °C as a result of EVA unsaturated backbone and PE hydrocarbon chains decomposition, leaving no residue at 800 °C [33,34]. The presence of ATH is responsible for an anticipated degradation due to water release, and the formation of an inorganic barrier that partially slows down the E-PE decomposition, as observed in the dTG curves (Figure 6 and Figure S4). The final residue consists of aluminum oxide, and increases as the ATH loading increases. TRZ and clay do not substantially modify this behavior likely due to the lower content with respect to ATH, as confirmed in samples containing different amounts of TRZ and clay (Figures S5 and S6). 

Under thermo-oxidative conditions, EVA de-acylation still occurs within the first decomposition step. Subsequently, the presence oxygen results in two separate weight loss steps in the 350–470 °C range related to the different thermo-oxidation of EVA and PE that produces a 10 wt.% residue, eventually oxidized above 500 °C (Figure 6C,D) [35]. The barrier produced by ATH limits oxygen diffusion and results in delayed and reduced degradation kinetics above 350 °C. This effect is clearly visible in the TG and dTG curves of Figure 6C,D. The presence of TRZ and clay improve the efficiency of the produced barrier as observable in the residues at 450 °C (i.e., 51%, 59% and 62%, for E-PE/120ATH, E-PE/120ATH/10TRZ and E-PE/120ATH/10TRZ/5Clay, respectively). TRZ is effective only at 15 and 20 phr, whereas different clay content does not change this effect, as reported in Figures S5 and S6.

### 3.4. Flammability

**Figure 7:**
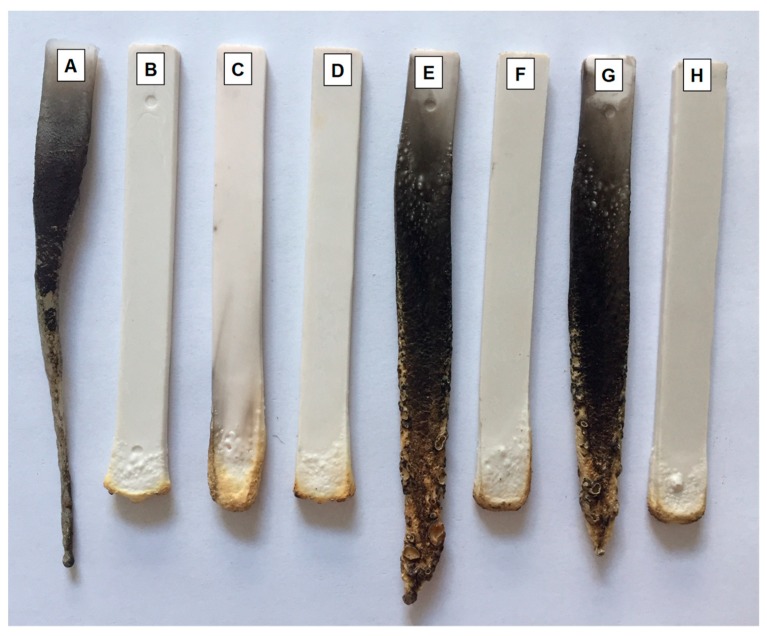
Digital pictures of specimens after UL94-V tests: (**A**) E-PE, (**B**) E-PE/185ATH, (**C**) E-PE/120ATH, (**D**) E-PE/120ATH/20TRZ, (**E**) E-PE/120ATH/15TRZ, (**F**) E-PE/120ATH/15TRZ/3clay, (**G**) E-PE/120ATH/10TRZ, and (**H**) E-PE/120ATH/10TRZ/5clay composites.

The flammability of E-PE blend and the investigated composites using ATH, TRZ additives and modified bentonite was assessed by UL94 vertical classification. This test evaluates the reaction of prepared materials when subjected to a direct flame application, thus providing information on their ability to start a fire. Table 3 reports UL94-V classification of the investigated materials and Figure 7 collects digital images of some specimens at the end of the test.

As is well-known, the E-PE blend is a highly flammable material. Indeed, upon flame application the sample starts to burn vigorously with the formation of flaming droplets that may extinguish the flame prior to the complete combustion of the sample, as reported in Figure 7A. This is highly undesirable as this phenomenon can easily spread the fire to other ignitable materials in a real fire scenario, thus resulting in a serious fire threat. The inclusion of ATH considerably changes the burning behavior of the composite (Figure 7B,C). Indeed, at 185 phr no ignition is observed after the first flame application while the second flame application results in short burning times (<5 s) granting the maximum rating for this test: V0 classification (Figure 7A). This is due to the formation of a protective inorganic barrier as ATH accumulates on the surface of the specimen exposed to the flame; the release of water also provides beneficial effects lowering the temperature of the flame and diluting volatiles. Reducing the content of ATH to 160 and 120 phr (Figure 7C) compromises the performances of the composites with a downgrade to not classifiable rating due to the presence of melt dripping. These results clearly confirm the mandatory need for very high ATH content in order to achieve good flame retardant effects, as already reported in the literature. To overcome this problem, TRZ and clay have been added to the formulation containing 120 phr of ATH (Figure 7D,E). The inclusion of 20 phr of TRZ alone allows for the maximum rating pairing the results of the E-PE/185ATH composites (see Figure 7D and Table 3). A reduction to 15 and 10 phr does not grant similar performances and results in extensive melt dripping (Figure 7E,G) and prolonged burning times (>60 s). Such results are improved by the addition of clay at either 3 or 5 phr, achieving the highest rating with TRZ at both 15 and 10 phr (Figure 7F,H). It is worth highlighting the beneficial role of modified bentonite that is capable of considerable improvements in the flame-retardant performances at relatively low loadings (i.e., E-PE/120ATH/10TRZ/5clay). Such results can be related to the good distribution and dispersion of the clay during processing. This is deemed to have a fundamental role in the achieved flame retardancy properties, as it allows the clay to substantially improve the efficiency of the barrier produced by ATH [7,36]. From the above results, the TRZ/clay combination allows for substantial reductions in the total filler loadings while maintaining fire safety.

### 3.5. Burning Behavior under Forced Flaming Combustion

**Figure 8:**
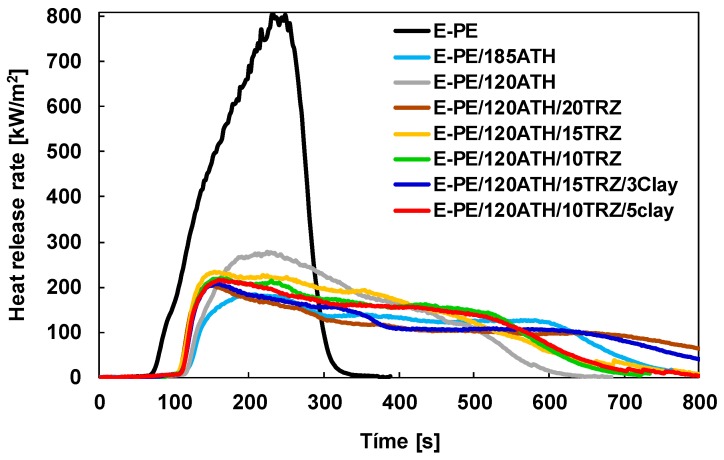
Heat release rate curves as a function of time for: E-PE, E-PE/185ATH, E-PE/120ATH, E-PE/120ATH/20TRZ, E-PE/120ATH/15TRZ, E-PE/120ATH/15TRZ/3clay, E-PE/120ATH/10TRZ, and E-PE/120ATH/10TRZ/5clay composites.

Cone calorimetry was employed to evaluate the reaction of prepared composites to the exposure to a heat flux typical of developing fires (i.e., 35 kW/m^2^). For this test, samples have been selected on the basis of flammability results and total filler loading in order to test the more efficient formulations along with their reference material. During the test, as a consequence of the heat flux exposure, the sample starts degrading and releasing flammable volatiles that are ignited by a spark positioned above the samples. Once ignition occurs, the instrument evaluates all parameters linked to heat and smoke release. The main parameter is the heat release rate, which as function of time, is reported in Figure 8. Table 4 collects the complete set of parameters for each composite.

An apparent flame-retardant effect can be achieved by including 185 phr of ATH with considerable reduction in heat release values (pkHRR, THR and MARHE reduced by 78%, 30% and 70%, respectively) as well as smoke production (TSR reduced by 52%). This result is related to both the reduced amount of polymer matrix in the composite and to the barrier and water release effect produced by ATH [37]. The produced barrier, clearly visible from the digital pictures of the residues reported in Figure S7, hinders volatile release and limits heat transmission and mass transfer from the flame to the polymer, resulting in reductions of combustion kinetics as well as smoke production. The released water can dilute smoke by reducing its optical density while simultaneously lowering the flame temperature. Reducing the content of the hydroxide to 120 phr maintains good flame retardant properties with the most apparent detrimental effect on TSR values likely due to the production of an inefficient barrier during combustion and to the lesser release of water. On the other hand, it should be pointed out that by reducing the ATH content, the amount of combustible polymer is inevitably increased, thus providing an additional challenge for the developed formulations.

As observed from flammability results, the inclusion of TRZ helps in improving the properties of the 120 phr ATH formulation from the assessment of pkHRR and TSR reductions. This can be ascribed to the mode of action of TRZ that, as for other melamine derivatives, shows mostly diluting and cooling effects in the gas phase [38]. The beneficial effect of TRZ is remarkable only at 20 phr while lower loadings only partially improve the pkHRR reduction (see Table 4). Further improvements of the formulations containing 15 and 10 phr of TRZ can be achieved by incorporating bentonite clay. The ability of clay in promoting the formation of a more efficient barrier to volatiles and heat transfer allows for further reducing pkHRR and TSR values [39,40], as reported in Table 4 mostly matching the results of E-PE/120ATH/20TRZ. The evaluation, by optical microscopy, of the top surface of the residues collected at the end of the test (Figure S8) show no apparent differences between each formulation. A compact and brittle inorganic layer mostly resulting from the cumulation of aluminum oxide at the polymer/flame interface is observed. The internal structure of the residue has also been investigated. To this aim, small pieces have been collected from the main structure and tilted in order to make the internal structure visible (Figure S9). Differences in macroscopic morphology can be easily detected. Indeed, while formulations containing 185 and 120 phr of ATH yielded a quite dense structure, the presence of TRZ produced porous structures with pore number and distribution proportional to TRZ content. This can be ascribed to the release of volatiles by TRZ and helps in improving the heat shielding properties of the produced protective layer that benefits from the reinforcing effect of clay [31,36]. From an overall point of view, the addition of TRZ and clay compensates for the reduced ATH content, thus providing a valuable strategy to simultaneously reduce the FR loading while guaranteeing considerable FR performances. Indeed, the observed reductions in combustion parameters (pkHRR, THR and MARHE reduced by 74%, 25% and 65%, respectively) and smoke production (TSR reduced by 32%), ensures the fire safety of the ATH/TRZ/clay formulations.

### 3.6. Mechanical Properties

The impact of the flame-retardant formulation on the mechanical properties of the E-PE blend was assessed by tensile tests [24]. Table 5 collects Young’s modulus, tensile strength, elongation at break and tenacity of prepared composites.

As is well-known, the high contents of FR additives needed to ensure safety inevitably result in substantial changes of the mechanical properties of the polymer matrix, increasing modulus and tensile strength while reducing elongation at break and tenacity [9,41]. Such behavior is observed for formulation containing 185 phr of ATH that show increased stiffness (Young´s modulus and tensile strength up to 92 and 13 MPa, respectively) and reduced deformability (elongation at break reduced from 478% to 101%). The reduction of ATH content from 185 to 120 phr partially limits this phenomenon (elongation at break is improved), but has the unwanted result of considerably limiting the fire safety of the prepared materials as demonstrated by flammability and cone testing. The inclusion of TRZ at 20 phr further increases the stiffness of the materials, while reducing its content to 15 and 10 phr partially improves the elongation at break and tenacity of the formulations with respect to E-PE/120ATH/20TRZ. Similarly, bentonite clay does not improve deformability with respect to the formulations containing ATH and TRZ. However, it should be pointed out that the elongation at break displayed by formulations containing TRZ and clay is always superior to E-PE/185ATH (i.e., 137% vs. 101%), which is the reference material as far as fire protection is concerned. Such improvements can be mainly ascribed to the reduced additive content. This indicates that composites such as E-PE/120ATH/15TRZ/3clay and E-PE/120ATH/10TRZ/5clay can improve on the overall deformability of the materials while still maintaining the required flame retardant properties. In order to evaluate the potential economic impact of the performed formulations, a simple cost-benefit analysis has been performed evaluating the cost of raw materials employed in the most performing formulations (Table 6).

It is apparent that the optimized E-PE/120ATH/10TRZ/5clay helps in saving up to 13% of the costs, with respect to the E-PE/185ATH reference sample (both to the same V0 classification according to the UL94 vertical configuration), thus making this formulation the most appealing from an industrial point of view.

## 4. Conclusions

In this work, melamine triazine and bentonite clay have been employed as novel flame-retardant additives for ethylene-vinyl acetate copolymer blends with polyethylene loaded with reduced amounts (i.e., 120 phr) of conventional aluminum trihydroxide particles. The aim was to maintain excellent flame-retardant properties, comparable with those of conventionally employed E-PE composites at high filler loading (i.e., 185 phr), while preserving mechanical properties. Different contents of TRZ and clay at fixed ATH content were prepared and thoroughly investigated from the morphology, rheology, thermal stability, flame retardancy and mechanical properties point of view. Optimized E-PE formulations grant self-extinguishing behavior during flammability tests in the vertical configuration, reaching the highest classification rating (V0) while E-PE/120ATH composites fail the test (not classifiable). The presence of TRZ and clay improves the efficiency of the protective barrier produced by ATH during combustion. This was also confirmed by cone calorimetry where samples containing TRZ and clay were capable of further reducing combustion kinetics (−23% in pkHRR) and smoke production (−11 % in TSR) with respect to the E-PE/120ATH reference. Mechanical properties showed significant improvements as compared with conventional formulations (i.e., E-PE/185ATH) with reduced stiffness and improved elongation at break. 

The ability of preserving mechanical properties while still achieving high flame-retardant performances in combination with easier processing conditions and reduced costs make the composites developed in this work highly promising and attractive solutions for further industrial exploitation.

## Figures and Tables

**Table 1:** Compositions of investigated composites.

Sample	ATH [phr] *	TRZ [phr]	Clay [phr]
E-PE	-	-	-
E-PE/185ATH	185	-	-
E-PE/160ATH	160	-	-
E-PE/120ATH	120	-	-
E-PE/120ATH/20TRZ	120	20	-
E-PE/120ATH/15TRZ	120	15	-
E-PE/120ATH/10TRZ	120	10	-
E-PE/120ATH/15TRZ/1CLAY	120	15	1
E-PE/120ATH/15TRZ/3CLAY	120	15	3
E-PE/120ATH/15TRZ/5CLAY	120	15	5
E-PE/120ATH/10TRZ/1CLAY	120	10	1
E-PE/120ATH/10TRZ/3CLAY	120	10	3
E-PE/120ATH/10TRZ/5CLAY	120	10	5

* phr = parts per hundred resin.

**Table 2:** Thermal data of polymer E-PE, E-PE/120ATH, E-PE/120ATH/10TRZ and E-PE/120ATH/10TRZ/5clay composites by thermogravimetric analyses.

Sample	Argon			Air				
	* T_max_ [°C]	Deriv. Mass [%/°C]	Residue at 800 °C [%]	* T_max 1_ [°C]	Deriv. Mass _1_ [%/°C]	* T_max 2_ [°C]	Deriv. Mass _2_ [%/°C]	Residue at 800 °C [%]
E-PE	467	2.27	0.0	346	0.67	410	1.69	1.5
E-PE/120ATH	472	1.02	37.2	320	0.35	385	0.48	40.2
E-PE/120ATH/10TRZ	476	1.13	35.2	318	0.31	470	0.42	32.4
E-PE/120ATH/10TRZ/5clay	476	1.06	34.8	315	0.33	467	0.49	34.9

* From derivative curves.

**Table 3:** Flammability results of E-PE blend and the investigated composites following the UL94 vertical configuration method.

Sample	t 1 ± σ	t 2 ± σ	UL94 Classification	Burning Characteristics
E-PE	>60	-	n.c. *	Intense melt dripping
E-PE/185ATH	-	3 ± 1	V0	No melt dripping
E-PE/160ATH	-	85 ± 26	n.c.	Moderated melt dripping
E-PE/120ATH	-	57 ± 35	n.c.	Flaming droplets
E-PE/120ATH/20TRZ	-	3 ± 1	V0	No melt dripping
E-PE/120ATH/15TRZ	41 ± 78	105 ± 30	n.c.	Intense melt dripping
E-PE/120ATH/10TRZ	7 ± 8	77 ± 40	n.c.	Intense melt dripping
E-PE/120ATH/15TRZ/1CLAY	-	7 ± 5	V1	No melt dripping
E-PE/120ATH/15TRZ/3CLAY	-	4 ± 2	V0	No melt dripping
E-PE/120ATH/15TRZ/5CLAY	-	4 ± 2	V0	No melt dripping
E-PE/120ATH/10TRZ/1CLAY	-	83 ± 39	n.c.	Intense melt dripping
E-PE/120ATH/10TRZ/3CLAY	-	9 ± 3	V1	No melt dripping
E-PE/120ATH/10TRZ/5CLAY	-	4 ± 3	V0	No melt dripping

* n.c.: not classifiable. Note: the occurrence of intense melt dripping in n.c. samples are responsible for a large standard deviation as this might cause the specimen to self-extinguish at random times.

**Table 4:** Combustion data results of E-PE and some corresponding composites by cone calorimetry.

Sample	TTI [s]	pkHRR [kW/m^2^]	THR [MJ/m^2^]	MARHE [kW/m^2^]	TSR [m^2^/m^2^]	Residue [%]
E-PE	62 ± 4	850 ± 59	110 ± 1	379 ± 15	1178 ± 33	0
E-PE/185ATH	111 ± 5	186 ± 2	77 ± 4	112 ± 2	570 ± 53	44 ± 1
E-PE/120ATH	107 ± 2	281 ± 14	80 ± 9	155 ± 5	907 ± 62	37 ± 1
E-PE/120ATH/20TRZ	98 ± 3	210 ± 5	88 ± 1	109 ± 4	725 ± 44	35 ± 1
E-PE/120ATH/15TRZ	99 ± 5	237 ± 13	86 ± 5	147 ± 9	951 ± 68	33 ± 1
E-PE/120ATH/10TRZ	103 ± 4	223 ± 15	83 ± 5	138 ± 10	945 ± 42	35 ± 1
E-PE/120ATH/15TRZ/3clay	101 ± 4	212 ± 12	86 ± 3	116 ± 3	843 ± 47	35 ± 1
E-PE/120ATH/10TRZ/5clay	101 ± 3	218 ± 9	82 ± 6	133 ± 2	806 ± 43	36 ± 1

**Table 5:** Mechanical properties of E-PE blend and corresponding composites.

Sample	Young’s Modulus [MPa]	Tensile Strength [MPa]	Elongation at Break [%]	Tenacity [MPa]
E-PE	27 ± 1	7.0 ± 0.3	478 ± 26	27 ± 2
E-PE/185ATH	92 ± 5	13.0 ± 0.4	101 ± 8	11 ± 1
E-PE/160ATH	68 ± 1	12.0 ± 0.2	147 ± 9	14 ± 1
E-PE/120ATH	58 ± 1	10.0 ± 0.2	165 ± 11	13 ± 1
E-PE/120ATH/20TRZ	58 ± 2	7.0 ± 0.4	115 ± 6	6 ± 1
E-PE/120ATH/15TRZ	61 ± 2	11.0 ± 0.1	180 ± 7	16 ± 1
E-PE/120ATH/10TRZ	68 ± 2	12.0 ± 0.4	167 ± 9	16 ± 1
E-PE/120ATH/15TRZ/1CLAY	69 ± 1	11.0 ± 0.2f	146 ± 11	13 ± 1
E-PE/120ATH/15TRZ/3CLAY	74 ± 3	11.0 ± 0.2	137 ± 4	12 ± 0
E-PE/120ATH/15TRZ/5CLAY	77 ± 2	11.0 ± 0.3	113 ± 5	10 ± 1
E-PE/120ATH/10TRZ/1CLAY	74 ± 2	12.0 ± 0.4	130 ± 9	13 ± 1
E-PE/120ATH/10TRZ/3CLAY	70 ± 2	12.0 ± 0.3	142 ± 6	13 ± 1
E-PE/120ATH/10TRZ/5CLAY	70 ± 1	10.0 ± 0.2	137 ± 8	11 ± 1

**Table 6:** Cost of the total loading for composite classified as V0 following the UL94 vertical configuration.

Sample	Formulation Composition	* Cost of Formulation/kg of E-PE [USD/kg]	* Cost of Formulation/m^3^ of E-PE [USD/m^3^]
E-PE/185ATH	185 phr ATH	7.3	6.8
E-PE/120ATH/20TRZ	120 phr ATH+20 phr TRZ	7.7	7.1
E-PE/120ATH/15TRZ/3clay	120 phr ATH+15 phr TRZ+3 clay	7.1	6.6
E-PE/120ATH/10TRZ/5clay	120 phr ATH+10 phr TRZ+5 clay	6.4	5.9

* Cost estimated for ATH: 3.95 USD/kg, TRZ: 15.00 USD/kg, clay: 5.00 USD/kg.

## Supplementary Materials

The following are available online at https://www.mdpi.com/1996-1944/12/15/2393/s1, Figure S1: Digital pictures of the screw configuration. 1-5 kneading element blocks, Figure S2: (A) Area of E-PE/120ATH composite analyzed by SEM for elemental analysis. (B) Aluminum mapping, Figure S3: TG and dTG curves of ATH, TRZ, and clay. (A,B) curves in argon, and (C,D) curves in air atmospheres, Figure S4: TG and dTG curves of E-PE/ATH composites varying ATH content. (A,B) curves in argon, and (C,D) curves in air atmospheres, Figure S5: TG and dTG curves of E-PE/120ATH/TRZ composites varying TRZ amount. (A,B) curves in argon, and (C,D) curves in air atmospheres, Figure S6: TG and dTG curves of E-PE/120ATH/15TRZ/clay and E-PE/120ATH/10TRZ/clay composites varying the content of modified bentonite. (A,B) curves in argon, and (C,D) curves in air atmospheres, Figure S7: Digital pictures of residues after cone calorimetry tests for: (A) E-PE, (B) E-PE/185ATH, (C) E-PE/120ATH, (D) E-PE/120ATH/20TRZ, (E) E-PE/120ATH/15TRZ, (F) E-PE/120ATH/15TRZ/3clay, (G) E-PE/120ATH/10TRZ, and (H) E-PE/120ATH/10TRZ/5clay composites. Figure S8: Optical microscopy pictures of the surface of the residues after cone calorimetry tests for: (A) E-PE/185ATH, (B) E-PE/120ATH, (C) E-PE/120ATH/20TRZ, (D) E-PE/120ATH/15TRZ, (E) E-PE/120ATH/10TRZ, (F) E-PE/120ATH/15TRZ/3clay, (G) E-PE/120ATH/10TRZ/5clay composites, Figure S9: Digital pictures of the internal portion of the residues after cone calorimetry tests for: (A) E-PE/185ATH, (B) E-PE/120ATH, (C) E-PE/120ATH/20TRZ, (D) E-PE/120ATH/15TRZ, (E) E-PE/120ATH/10TRZ, (F) E-PE/120ATH/15TRZ/3clay, (G) E-PE/120ATH/10TRZ/5clay composites. Table S1: Thermal data of ATH, melamine triazine (TRZ) and modified bentonite (clay) in argon and air atmospheres, Table S2: Thermal data of E-PE/ATH composites using different content of ATH by thermogravimetric analysis, Table S3: Thermal data of E-PE/120ATH/TRZ composites varying TRZ content by thermogravimetric analysis, Table S4: Thermal data of E-PE/120ATH/15TRZ/clay and E-PE/120ATH/10TRZ/clay composites varying bentonite content by thermogravimetric analysis.
