# Supplementary material for: Improving Mechanical Properties and Reaction to Fire of EVA/LLDPE Blends for Cable Applications with Melamine Triazine and Bentonite Clay
Source: Materials (Basel). 2019 Jul 26;12(15):2393. doi: 10.3390/ma12152393 (PMC6696111; doi:10.3390/ma12152393)
Supplement: Supplementary file 1 [file materials-12-02393-s001.pdf]

materials

MDPI

Article

Improving Mechanical Properties and Reaction to Fire of EVA/LLDPE Blends for Cable Applications with Melamine Triazine and Bentonite Clay

Guadalupe Sanchez-Olivares [1], Antonio Sanchez-Solis [2], Octavio Manero [2], Ricardo Pérez-Chávez [1], Mario Jaramillo [1], Jenny Alongi [3] and Federico Carosio [4,*]

1 CIATEC, A.C., Omega 201, Col. Industrial Delta, 37545 León, Gto., Mexico; gsanchez@ciatec.mx (G.S.-O.); rperez@ciatec.mx (R.P.-C.); ma.jaramillozuniga@ugto.mx (M.J.)
2 Instituto de Investigaciones en Materiales, Universidad Nacional Autónoma de México, Avenida Universidad 3000, 04510 Ciudad de México, Mexico; sancheza@unam.mx (A.S.-S.); manero@unam.mx (O.M.)
3 Dipartimento di Chimica, Università degli Studi di Milano, Via Golgi 19, 20133 Milano, Italy; jenny.alongi@unimi.it
4 Dipartimento di Scienza Applicata e Tecnologia, Politecnico di Torino, Alessandria Campus, Viale Teresa Michel 5, 15121 Alessandria, Italy
* Correspondence: federico.carosio@polito.it; Tel.: +39-0131-229303

Figure S1 shows digital images of twin-screw configuration. The screw configuration has five kneading element blocks, localized (from die to feeding zone) at: 1) 330 mm with 5 elements, each one of 7 mm wide; 2) 480 mm, with 8 elements, each one of 7 mm wide; 3) 650 mm, with 10 elements, each one of 7 mm wide; 4) 850 mm, with 4 elements, each one of 5 mm wide and 14 elements, each one of 7 mm wide and 5) a dispersive zone at 1150 mm, with 15 elements, each one of 7 mm wide.

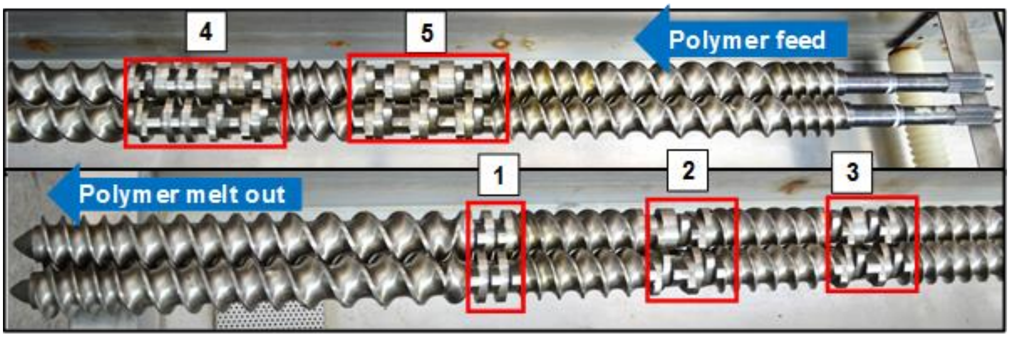

Figure 1. Digital pictures of the screw configuration. 1-5 kneading element blocks.

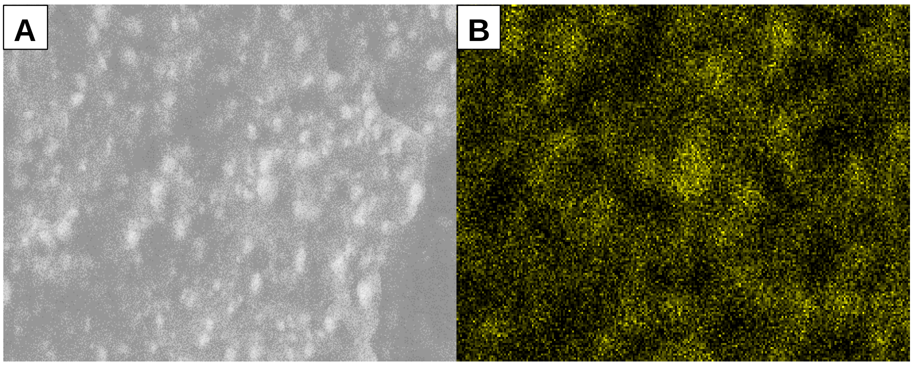

Figure 2. (A) Area of E-PE/120ATH composite analyzed by SEM for elemental analysis. (B) Aluminum mapping.

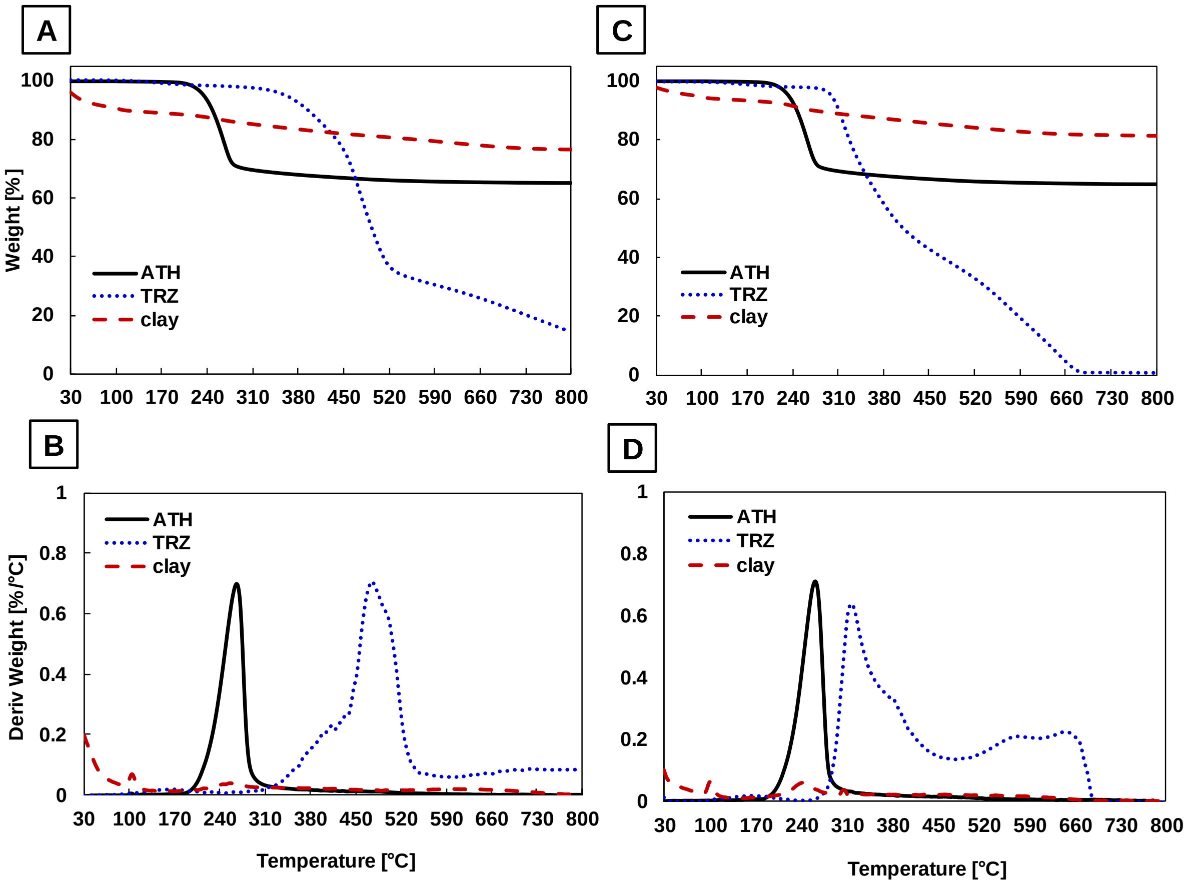

Figure 3. TG and dTG curves of ATH, TRZ, and clay. (A,B) curves in argon, and (C,D) curves in air atmospheres.

Table S1. Thermal data of ATH, melamine triazine (TRZ) and modified bentonite (clay) in argon and air atmospheres.

**Sample**	**Argon**			**Air**				
	***T_{max} [°C]**	**Deriv. mass [%/°C]**	**Residue at 800 °C [%]**	***$T_{max\ 1}$ [°C]**	**Deriv. mass $_1$ [%/°C]**	***$T_{max\ 2}$ [°C]**	**Deriv. mass $_2$ [%/°C]**	**Residue at 800 °C [%]**
ATH	266	0.70	65.2	262	0.71	-	-	65.0
TRZ	476	0.70	14.1	319	0.64	648	0.22	0.5
Clay	258	0.04	76.7	-	-	243	0.06	81.2

*From derivative curves.

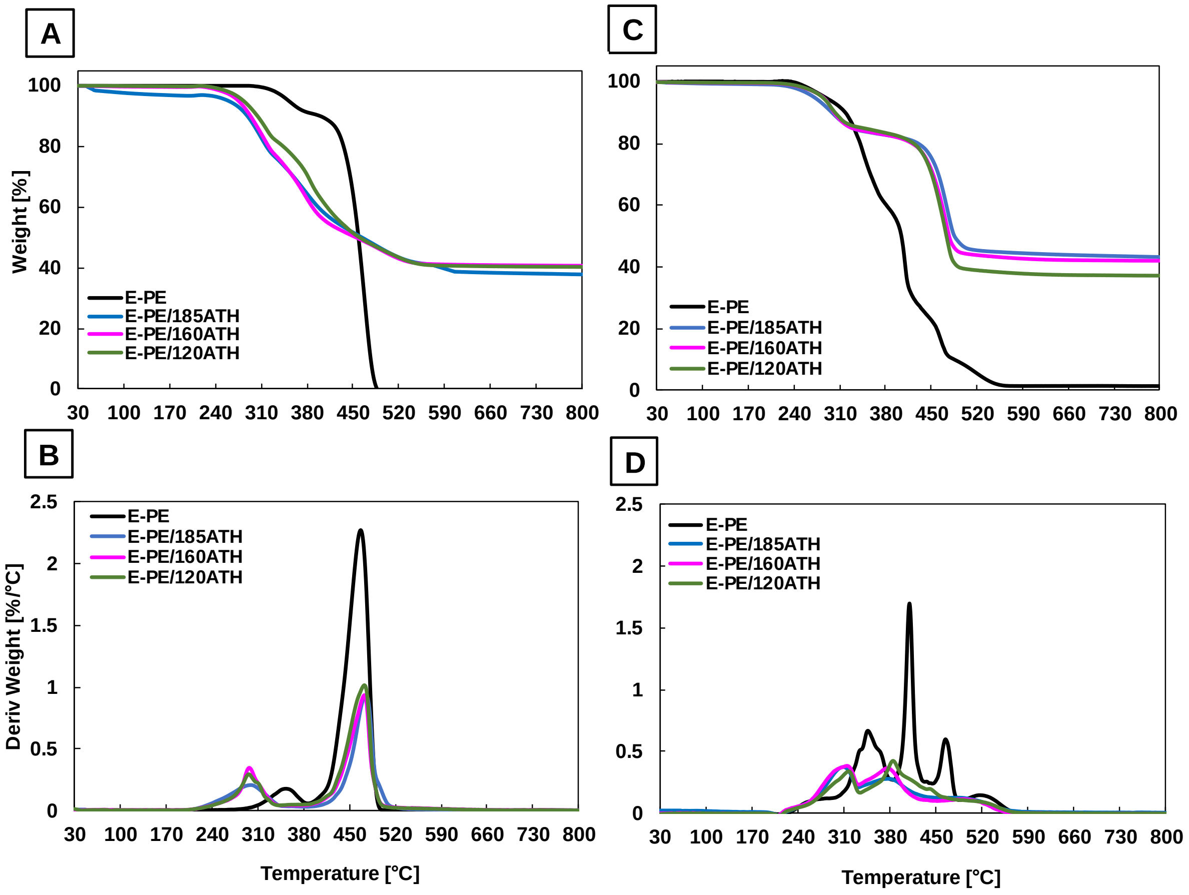

Figure S4. TG and dTG curves of E-PE/ATH composites varying ATH content. (A,B) curves in argon, and (C,D) curves in air atmospheres.

Table S2. Thermal data of E-PE/ATH composites using different content of ATH by thermogravimetric analysis.

**Sample**	**Argon**			**Air**				
	***T$_{max}$ [°C]**	**Deriv. mass [%/°C]**	**Residue at 800 °C [%]**	***T$_{max 1}$ [°C]**	**Deriv. mass $_1$ [%/°C]**	***T$_{max 2}$ [°C]**	**Deriv. mass $_2$ [%/°C]**	**Residue at 800 °C [%]**
E-PE	467	2.27	0.0	346	0.67	410	1.69	1.5
E-PE/185ATH	474	0.93	43.4	308	0.37	377	0.28	39.8
E-PE/160ATH	472	0.94	42.2	312	0.38	377	0.36	40.6
E-PE/120ATH	472	1.02	37.2	320	0.35	385	0.48	40.2

*From derivative curves.

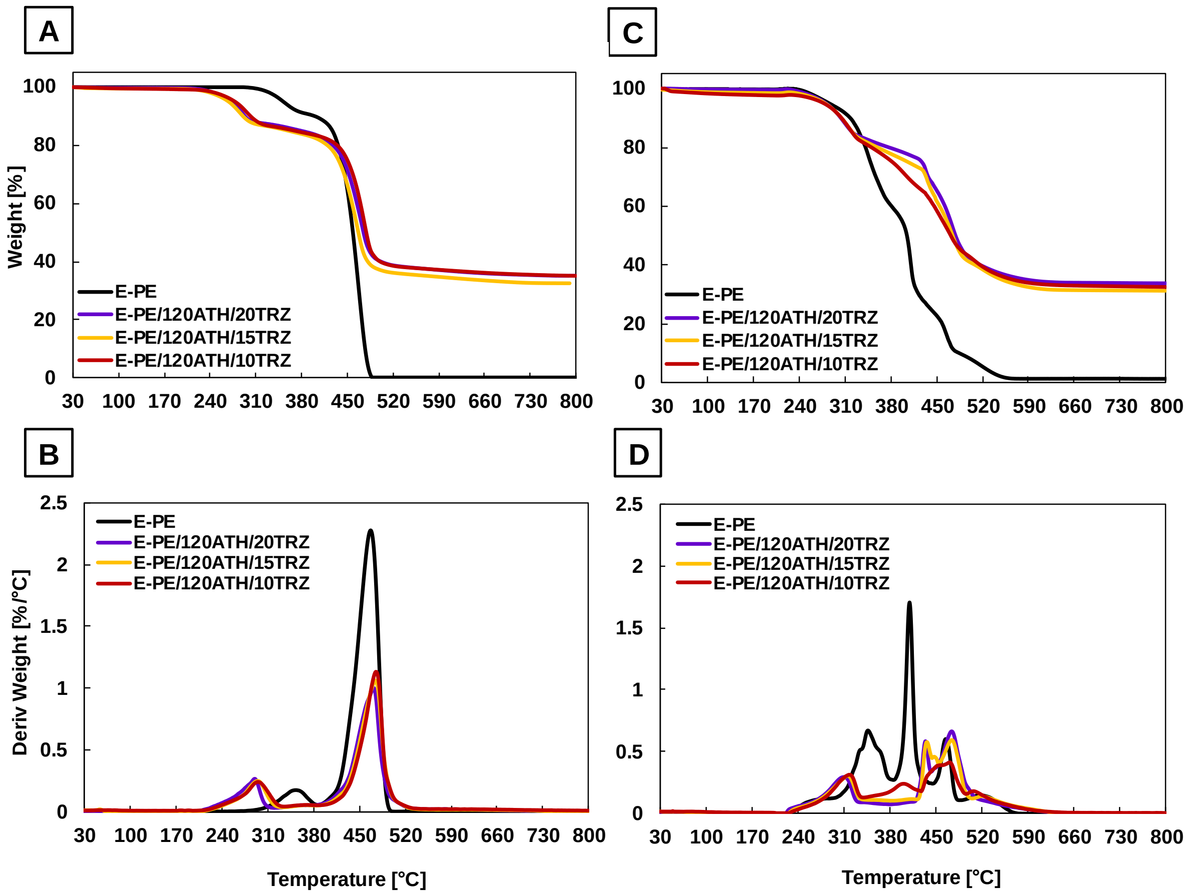

Figure S5. TG and dTG curves of E-PE/120ATH/TRZ composites varying TRZ amount. (A,B) curves in argon, and (C,D) curves in air atmospheres.

Table S3. Thermal data of E-PE/120ATH/TRZ composites varying TRZ content by thermogravimetric analysis.

Sample	Argon			Air				
	*T_{max} [°C]	Deriv. mass [%/°C]	Residue at 800 °C [%]	*$T_{max\ 1}$ [°C]	Deriv. mass 1 [%/°C]	*$T_{max\ 2}$ [°C]	Deriv. mass 2 [%/°C]	Residue at 800 °C [%]
E-PE	467	2.27	0.0	346	0.67	410	1.69	1.5
E-PE/120ATH/20TRZ	472	1.01	35.1	434	0.58	473	0.66	33.8
E-PE/120ATH/15TRZ	474	1.07	31.5	437	0.57	474	0.59	30.1
E-PE/120ATH/10TRZ	476	1.13	35.2	318	0.31	470	0.42	32.4

*From derivative curves.

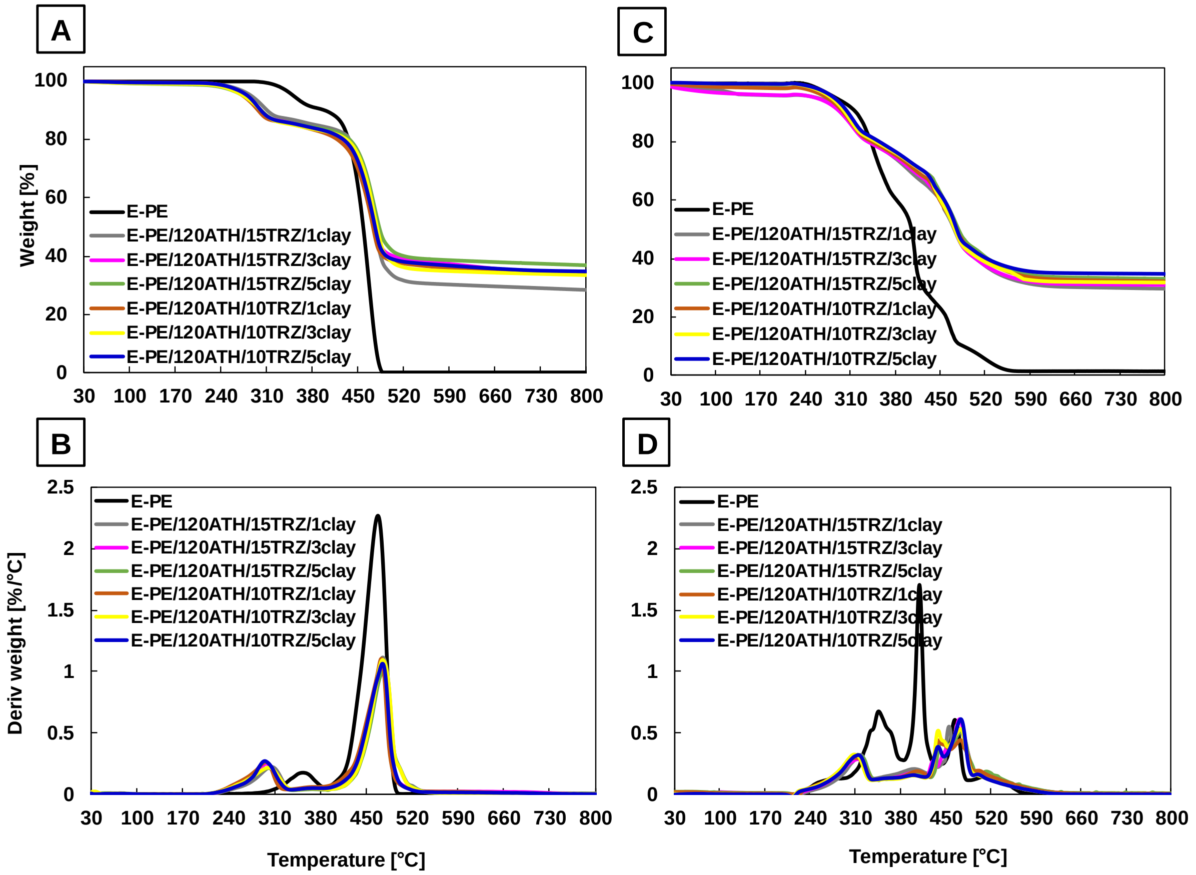

Figure S6. TG and dTG curves of E-PE/120ATH/15TRZ/clay and E-PE/120ATH/10TRZ/clay composites varying the content of modified bentonite. (A,B) curves in argon, and (C,D) curves in air atmospheres.

Table S4. Thermal data of E-PE/120ATH/15TRZ/clay and E-PE/120ATH/10TRZ/clay composites varying bentonite content by thermogravimetric analysis.

Sample	Argon			Air				
	*T_{max} [°C]	Deriv. mass [%/°C]	Residue at 800 °C [%]	*$T_{max\,1}$ [°C]	Deriv. mass $_1$ [%/°C]	*$T_{max\,2}$ [°C]	Deriv. mass $_2$ [%/°C]	Residue at 800 °C [%]
E-PE	467	2.27	0.0	346	0.67	410	1.69	1.5
E-PE/120ATH/15TRZ/1clay	475	1.12	28.6	455	0.54	473	0.51	29.8
E-PE/120ATH/15TRZ/3clay	474	0.89	33.9	316	0.28	471	0.61	30.8
E-PE/120ATH/15TRZ/5clay	474	0.99	36.8	314	0.32	474	0.50	33.0
E-PE/120ATH/10TRZ/1clay	476	1.08	33.8	439	0.43	472	0.43	32.4
E-PE/120ATH/10TRZ/3clay	476	1.09	33.7	316	0.27	476	0.51	31.7
E-PE/120ATH/10TRZ/5clay	476	1.06	34.8	315	0.33	467	0.49	34.9

*From derivative curves.

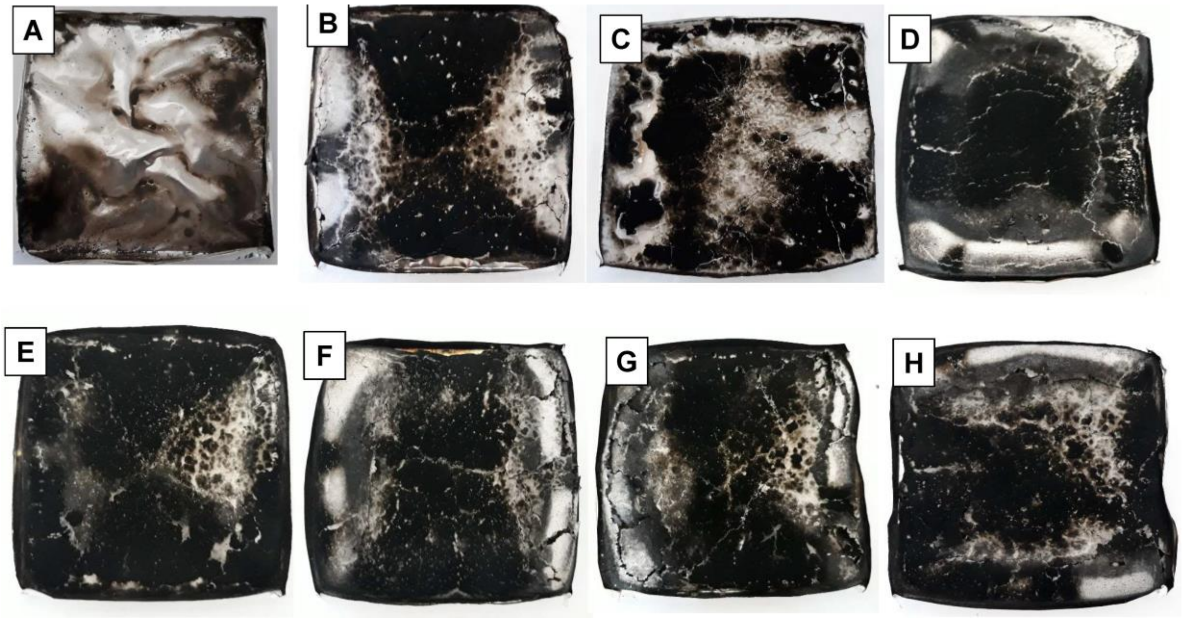

Figure S7. Digital pictures of residues after cone calorimetry tests for: (A) E-PE, (B) E-PE/185ATH, (C) E-PE/120ATH, (D) E-PE/120ATH/20TRZ, (E) E-PE/120ATH/15TRZ,(F) E-PE/120ATH/15TRZ/3clay, (G) E-PE/120ATH/10TRZ, and (H) E-PE/120ATH/10TRZ/5clay composites.

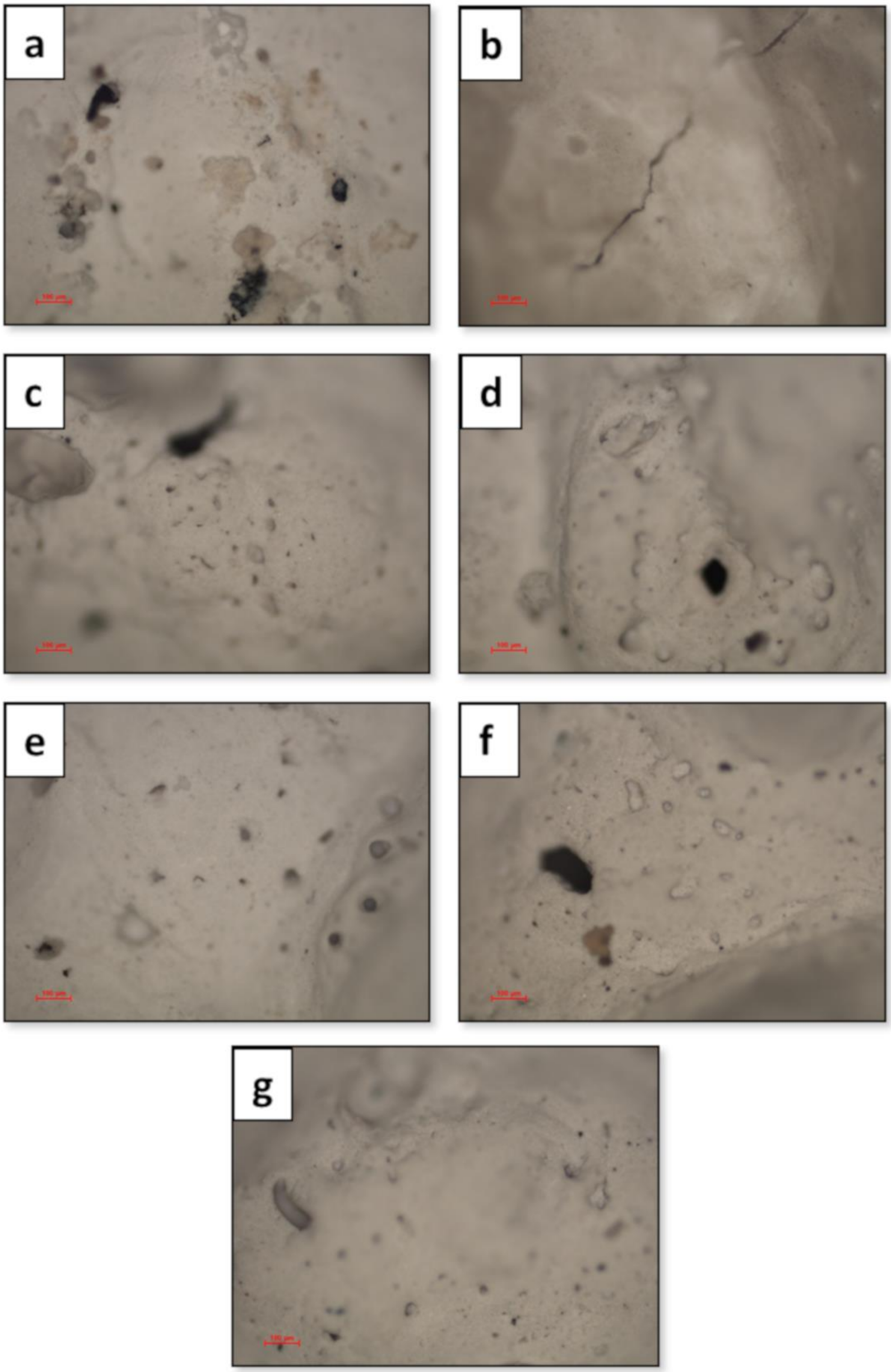

Figure S8. Optical microscopy pictures of the surface of the residues after cone calorimetry tests for: (A) E-PE/185ATH, (B) E-PE/120ATH, (C) E-PE/120ATH/20TRZ, (D) E-PE/120ATH/15TRZ, (E) E-PE/120ATH/10TRZ, (F) E-PE/120ATH/15TRZ/3clay, (G) E-PE/120ATH/10TRZ/5clay composites.

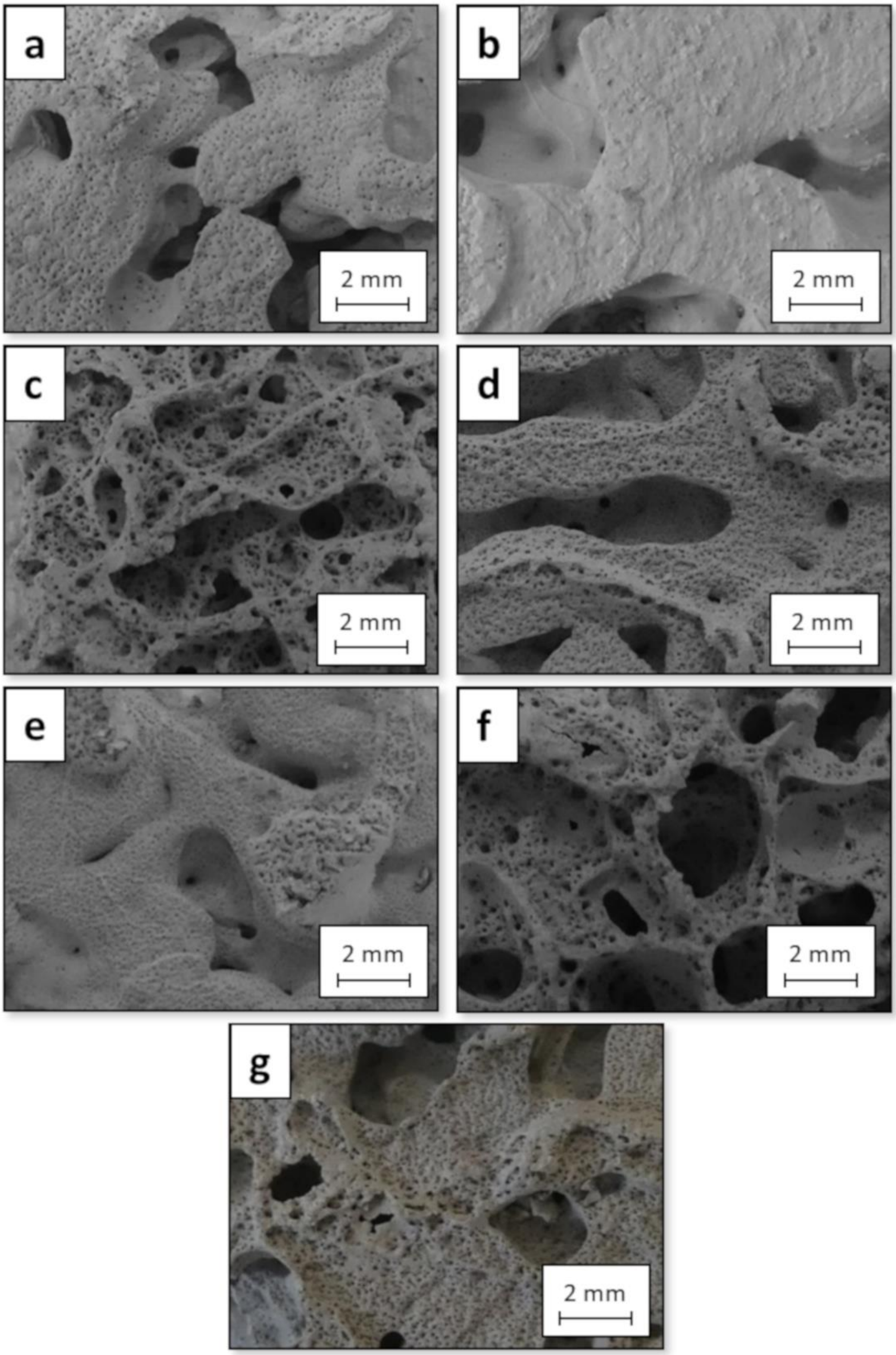

Figure S9. Digital pictures of the internal portion of the residues after cone calorimetry tests for: (A) E-PE/185ATH, (B) E-PE/120ATH, (C) E-PE/120ATH/20TRZ, (D) E-PE/120ATH/15TRZ, (E) E-PE/120ATH/10TRZ, (F) E-PE/120ATH/15TRZ/3clay, (G) E-PE/120ATH/10TRZ/5clay composites.
